# Whole Genome Distribution and Ethnic Differentiation of Copy Number Variation in Caucasian and Asian Populations

**DOI:** 10.1371/journal.pone.0007958

**Published:** 2009-11-23

**Authors:** Jian Li, Tielin Yang, Liang Wang, Han Yan, Yinping Zhang, Yan Guo, Feng Pan, Zhixin Zhang, Yumei Peng, Qi Zhou, Lina He, Xuezhen Zhu, Hongyi Deng, Shawn Levy, Christopher J. Papasian, Betty M. Drees, James J. Hamilton, Robert R. Recker, Jing Cheng, Hong-Wen Deng

**Affiliations:** 1 School of Medicine, University of Missouri Kansas City, Kansas City, Missouri, United States of America; 2 The Key Laboratory of Biomedical Information Engineering of Ministry of Education and Institute of Molecular Genetics, School of Life Science and Technology, Xi'an Jiaotong University, Xi'an, Shanxi, People's Republic of China; 3 Vanderbilt Microarray Shared Resource, Vanderbilt University, Nashville, Tennessee, United States of America; 4 Osteoporosis Research Center, Creighton University, Omaha, Nebraska, United States of America; 5 National Engineering Research Center for Beijing Biochip Technology, Changping District, Beijing, People's Republic of China; 6 Laboratory of Molecular and Statistical Genetics, College of Life Sciences, Hunan Normal University, Changsha, Hunan, People's Republic of China; Innsbruck Medical University, Austria

## Abstract

Although copy number variation (CNV) has recently received much attention as a form of structure variation within the human genome, knowledge is still inadequate on fundamental CNV characteristics such as occurrence rate, genomic distribution and ethnic differentiation. In the present study, we used the Affymetrix GeneChip® Mapping 500K Array to discover and characterize CNVs in the human genome and to study ethnic differences of CNVs between Caucasians and Asians. Three thousand and nineteen CNVs, including 2381 CNVs in autosomes and 638 CNVs in X chromosome, from 985 Caucasian and 692 Asian individuals were identified, with a mean length of 296 kb. Among these CNVs, 190 had frequencies greater than 1% in at least one ethnic group, and 109 showed significant ethnic differences in frequencies (*p*<0.01). After merging overlapping CNVs, 1135 copy number variation regions (CNVRs), covering approximately 439 Mb (14.3%) of the human genome, were obtained. Our findings of ethnic differentiation of CNVs, along with the newly constructed CNV genomic map, extend our knowledge on the structural variation in the human genome and may furnish a basis for understanding the genomic differentiation of complex traits across ethnic groups.

## Introduction

Variation within the human genome can take many different forms. One form of structural variation is copy number variation (CNV), in which a DNA segment, ranging from 1 kb to several megabases, is present at a variable copy number in comparison to a reference genome [1]. CNVs are widespread in the human genome, and vary across populations with respect to rate of occurrence [2]–[7]. CNVs have been shown to account for nearly 18% of variation in gene expression and, consequently, may play an important role in determining complex traits [8]. CNVs have been associated with certain complex human diseases, such as susceptibility to HIV infection, selected autoimmune diseases, tumors and psychiatric disorders such as mental retardation and autism [9]–[14].

Although several studies have been performed to characterize genomic CNVs, comparing results from these studies has been hindered by small sample sizes and different study designs and analytical methods. Consequently, it has been difficult to combine results from different studies to produce an accurate description of genomic CNV characteristics such as the total number, genomic position, gene content, and frequency distribution [7]. It is even more difficult to robustly detect CNV differentiation across ethnic groups, and this has limited the utility of CNVs for association studies and human evolution research. One approach that can minimize the problems listed above is to use large sample sizes comprised of subjects from comparatively homogeneous ethnic backgrounds for each study population [15]. Recent technologic developments such as the availability of high-density SNP microarrays have also been helpful, in terms of providing an efficient and affordable tool for CNV discovery in the human genome.

In this study, we utilized the Affymetrix GeneChip® Mapping 500K Array, in which one SNP was placed approximately every 5.8 kb along the human genome, to identify CNVs in both a US Caucasian population and a Chinese Han population. CNVs were identified and characterized based on probe intensities and SNP genotypes, and their ethnic differences were studied. The results extend our understanding on the structural variation in the human genome and may furnish a basis for understanding the genomic differentiation of complex traits across ethnic groups.

## Results

Brief summaries of CNV and CNVR (copy number variation region, which is a region covered by overlapping CNVs) characteristics in each ethnic group were shown in Table 1, with detailed summaries being presented in the corresponding supplementary tables.

**Table 1:** Characteristics of CNVs and CNVRs detected in Caucasian population and in Chinese population.

	Autosomes		X chromosome	
	CAU	CHI	CAU	CHI
CNVs				
Total # of CNV calls	9,196	6,858	1,318	1,232
Total # of CNV calls with gain	4,748	3,465	609	497
Total # of CNV calls with loss	4,448	3,393	709	735
Total # of CNVs	1,352	1,395	371	346
Mean size of CNVs	295 kb	303 kb	299 kb	277 kb
Median size of CNVs	195 kb	196 kb	202 kb	193 kb
CNVRs				
Total # of CNVRs	593	633	99	116
Mean size of CNVRs	361 kb	345 kb	493 kb	418 kb
Median size of CNVRs	229 kb	218 kb	242 kb	214 kb
Mean # of Probes per CNVR	58	52	36	30
Median # of Probes per CNVR	38	35	20	17
Genome coverage by CNVRs	215 Mb	218 Mb	49 Mb	39 Mb
Genome coverage per individual	2.1 Mb	3.3 Mb	192 kb	255 kb

### Characteristics of CNVs

**Figure 1:**
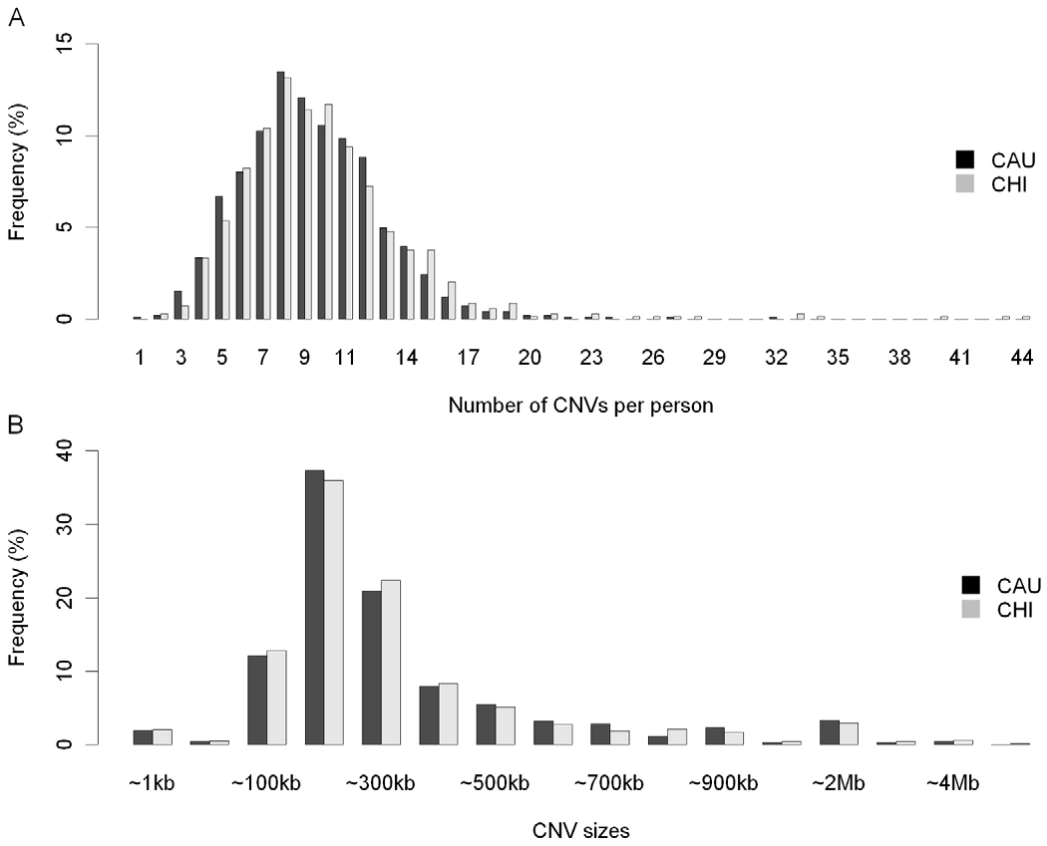
Characteristics of CNVs on autosomes. (A) Distribution of CNV numbers per individual. (B) Distribution of CNV sizes.

There were 2,381 autosomal CNVs identified in the 1,677 subjects (**Table S1**), with a median length of 198 kb and a mean length of 298 kb. Although CHI had a smaller sample size, the numbers of CNVs identified in the two ethnic groups were similar: 1,352 CNVs in CAU versus 1,395 CNVs in CHI. Other CNV characteristics that were similar in the two populations include the average number of CNVs per individual (∼9 CNVs per individual, ranging from 1–32, in CAU versus ∼10 CNVs per individual, ranging from 2–44 in CHI (**Figure 1A**), the median size of CNVs (195 kb in CAU vs. 196 kb in CHI), and the mean size of CNVs (295 kb in CAU vs. 303 kb in CHI) (**Figure 1B**). Although a great percentage of CNVs were singletons, 27.6% were present more than once in our samples. Specifically, 168 or 7% of the 2,381 CNVs were “common” CNVs, defined as CNVs with a frequency of 1% or greater in at least one ethnic group (**Table S2**).

There were 638 CNVs identified on the X chromosome in our subjects (**Table S1**), with a median length of 206 kb and a mean length of 288 kb, similar to those of autosomal chromosomes. For these 638 CNVs, 183 (29%) were detected in more than one subject, and 21 (3.6%) were common CNVs (**Table S2**). Most individuals in both populations had 3 or less CNVs on the X chromosome.

**Figure 2:**
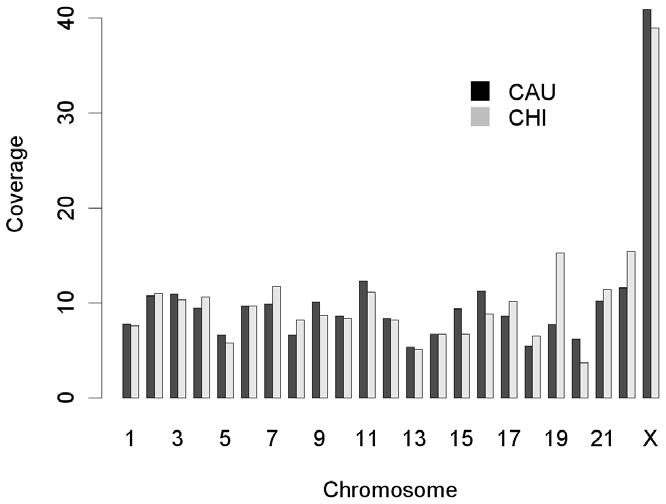
CNVR chromosomal coverage in CAU and in CHI. The vertical axis is the proportion of a specific chromosome covered by CNVRs. The proportion ranges from ∼3% to ∼15% in CHI and from ∼5% to ∼11% in CAU on autosomes and an extensive difference on X chromosome.

There were 593 autosomal CNVRs obtained in CAU, covering approximately 215 Mb (7.5%) of 22 autosomes with a median length of 229 kb and a mean length of 361 kb (ranging from 3 kb to ∼6 Mb). In CHI, 633 CNVRs covering approximately 218 Mb (7.6%) of 22 autosomes were obtained, with slightly shorter median and mean lengths (218 kb and 345 kb, respectively) compared to CAU, and ranging from 3 kb to ∼5 Mb. In total, 985 CNVRs covering ∼366 Mb (12.8%) of 22 autosomes were detected (**Table S3**). These CNVRs covered 59,936 probes, with a median of 39 probes and a mean of 61 probes covered by a single CNVR. CNVR coverage was similar when ethnic groups were considered separately, with increased coverage on chromosomes 19 and 22 in CHI (**Figure 2**).

A count of all CNVs occurring in the same CNVR was used to describe CNV occurrence rate in this CNVR. Only 97 (10%) of the CNVRs showed CNV occurrence rates higher than 1% in at least one population (**Table S4**). In addition, CNVRs with gains were more abundant than those with losses (463 with gains versus 365 with losses). The length of CNVRs with losses was found to be similar to the lengths of CNVRs with gains (305 kb versus 311 kb).

**Figure 3:**
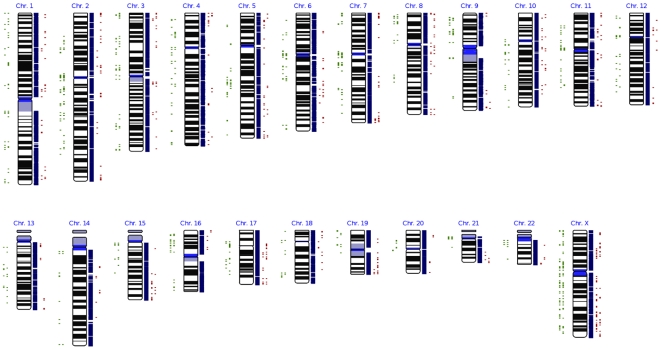
Genomic map for CNVs found in CAU and in CHI. The figure was drawn by IdeogramBrowser [28]. Individual chromosomes are shown by the karyograms, with dots on the left and right sides of the karyograms indicating chromosomal locations of copy number losses and gains, respectively. For dots in the two columns on the same side of the karyograms, those closer to the karyograms represent CNVs detected in CAU, and those further away indicate CNVs detected in CHI. Blue bars with white space on the right side of the karyograms indicate locations of known genes, based on the database available when the analyses were performed.

Ninety-nine CNVRs covering approximately 49 Mb (32%) of the X chromosome were obtained in CAU, with a median length of 248 kb and a mean length of 496 kb; 50% of these 99 CNVRs contained more than one CNV. In CHI, 116 CNVRs were obtained, covering approximately 39 Mb (26%) of the X chromosome. Compared to CAU, CHI CNVRs on the X chromosome had slightly shorter median and mean lengths (209 kb and 339 kb, respectively), and 54 of these CNVRs contained more than one CNV. In total, 150 CNVRs covering approximately 74 Mb (49%) of the X chromosome were detected (**Table S3 and** **Figure 2**). The X chromosome was covered by 5,485 probes, and a single CNVR was covered by a median of 24 probes and a mean of 37 probes. Twenty-eight of the 150 CNVRs (∼21%) detected had CNV occurrence rates greater than 1% (**Table S4**). Genomic map for CNVs found in CAU and in CHI was shown in **Figure 3**.

### Ethnic Differentiation of CNVs

Among the 2,381 autosomal CNVs, 15.4% were detected in both populations, 41.4% only in CAU, and 43.2% only in CHI. The number of CNVs on the X chromosome identified in both ethnic groups, in CAU only, and in CHI only, were 79 (12.4% of 638), 292 (45.8%), and 267 (41.8%), indicating significant ethnic differences in occurrence of genomic CNVs. The average coverage of autosomal CNVs in a CHI individual was 3.3 Mb, 1.6-fold higher than that observed in CAU (2.1 Mb). The autosomal coverage of gains and losses in CHI were 1.8-fold and 1.3-fold, respectively, higher than those in CAU. The average individual genomic coverage of X-chromosomal CNVs was 192 kb in CAU and 255 kb in CHI. Genomic coverage of X-chromosomal CNVs with gains and losses in CHI were 1.4-fold and 1.2-fold higher than those observed in CAU, respectively. As higher genomic coverages of CNVs were shown in a number of samples, mainly Chinese, a sensitivity analysis was performed by excluding all samples with a genomic coverage above 0.3 independent from the ethnicity. This resulted in 1.2-fold genomic coverage in CHI as in CAU.

Ninety-three (55% of 168) common autosomal CNVs and 16 (76% of 21) common X CNVs showed significant ethnic differences in frequencies (*p*<0.01) (**Table S2)**. Of the 109 ethnic different CNVs, 9 were identified only in CHI and 16 only in CAU. Since the CNV analysis cannot reveal the relationship of overlapping CNVs, we also perform the ethnic difference analysis of CNVRs. We found some “hot” genomic regions with ethnic differentiation in CNV occurrence rates (*p*<0.01) (**Table S4**).

Samples were clustered into the correct group with high possibility (>99%) when 2,000 unlinked SNPs were used to detect the population substructure, indicating our samples were homogeneous within each ethnic group and significant differences in CNVs are unlikely due to population mixture.

### CNV-Associated Genes

**Figure 4:**
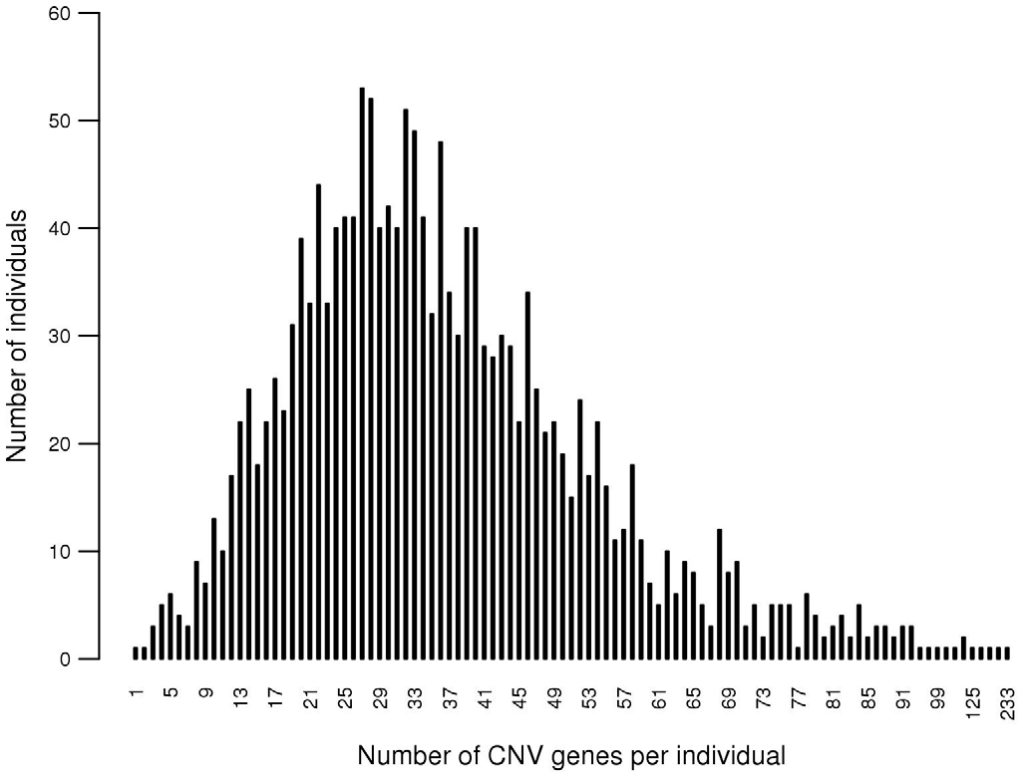
Distribution of CNV genes in 1677 individuals.

Genes overlapped, at least partially, with the identified CNVRs were defined as CNV-associated genes or CNV genes. 1737 CNV genes were identified from 985 Caucasians, with an average number of 35±17 (mean±SD) per individual. 2007 CNV genes were identified from 692 Asians, with an average number of 39±21 per individual. Totally 2796 genes were identified overlap with CNVRs in our samples, with an average number of 37±19 per individual, ranging from 1–233 (**Figure 4**). 88.4% of 1677 individuals had CNV genes ranging from 10 to 60. 15.7% of 2796 genes had CNV occurrence rates greater than 1%.

317 (13.6% of 2329) genes involve 392 genetic diseases in the OMIM (Online Mendelian Inheritance in Man) database in our results, which mainly related with mental or neural disorders, sensory disorders, cancers and so on. 13 CNV genes were related with mental retardation, 6 related with obesity, 5 related with schizophrenia and 3 with autism. Other diseases such leukemia, blood group and deafness were also identified affected by CNV genes. The majority of these disease-overlapping CNVRs involved low CNV occurrence rates (<1%). For autosomal CNVRs, 15 in CAU and 17 in CHI were detected with CNV occurrence rates greater than 1%, and these overlapped with 28 and 22 disease loci, respectively. On the X chromosome, the numbers of common CNVRs were 11 in CAU and 11 in CHI, respectively, overlapping with 40 and 39 OMIM genes (**Table S5**). This suggests the potential significance of these CNVRs in the etiology of human diseases.

Further analysis showed that 261 (9.4% of 2796) genes had significant ethnic differentiation (*P*<0.05) of CNV occurrence rates between CAU and CHI. We found 30 (11.5% of 261) CNV genes affect 46 human genetic diseases including blood group, deafness, mental retardation, Cohen syndrome, leukemia, obesity and Parkinson disease (**Table S6**).

### Novel CNVRs

Compared with the Database of Genomic Variants updated on September 5, 2007 [16], 69% (680) of our 985 autosomal CNVRs overlapped with previously published CNVs. The remaining 305 CNVRs were novel and covered 2.5% (72.1 Mb) of the 22 autosomes. Of the 305 novel CNVRs, 60 were detected in more than one individual, and five (region_14, region_197, region_325, region_618 and region_891) had CNV occurrence rates greater than 1%. For the 150 CNVRs detected on the X chromosome, 41% or 62 were novel CNVRs and covered 15% (22.1 Mb) of the X chromosome. Among the novel X CNVRs, 33 were detected in more than one individual, 8 were common CNVRs (**Table S3**).

## Discussion

Using the Affymetrix Genechip® 500K array, we have identified a large number of CNVs and CNVRs in US Caucasian and Chinese Han populations. CNVs are widespread in the human genome and cover about 8% of the genome in each ethnic group, with only ∼15% overlapping between CAU and CHI. We find that CNVs exhibit differences in occurrence and in frequency both at the individual level and at the population level. Our results showed that different ethnic groups bear different CNVs, similar to findings for microsatellites and SNPs, and CNV characteristics, such as frequencies, can differ across ethnic groups.

The description of CNV characteristics can be complicated. For example, CNVs in the same region can be of different types, have different breakpoints and variable CN changes among individuals. CNVs can overlap with other CNVs, can be nested within other CNVs, or can be completely separate from other CNVs. In region_571 (chr9: 42,525,734…44,108,554), we identified 20 CNVs with different frequencies (6%–13%) in both ethnic groups, with nearly half the samples showing CN change and some having three CNVs in this region. Another example is that the CNV occurrence rates in the same CNVR vary between populations. Most CNVs and CNVRs with frequencies or CNV occurrence rates greater than 1% showed significant ethnic differences. Nearly 85% of detected CNVs occur in only one specific ethnic group (e.g. CNV_0132 and CNV_0421) and 15% in both ethnic groups, often with different frequencies (e.g. CNV_0115, CNV_0553, CNV_1104 and CNV_1267). A third example of ethnic variation is that the genomic coverage of CNVs in Asians is 1.6-fold higher than that in seen in Caucasians, indicating that genome variants were more common in Asians than in Caucasians. Further sensitivity analysis resulted in an only 1.2-fold elevated genomic coverage of Chinese CNVs compared to Caucasian CNVs. Considering different DNA extraction methods for Chinese and Caucasian samples, the ethnic differences seen in genomic coverage may be partially due to different binding affinities of diverse DNA extracts to the SNP chip array.

Increasing evidence shows that CNVs can affect gene expression for genes related to complex traits, but little is known on how CNVs can cause phenotypic diversity. A recent study showed that CNVs account for 17.7% of detected genetic variation in gene expression [8]. This percentage may be an under-estimate, since it is unlikely that current CNV detection technology completely reveals the whole genome CNVs. Furthermore, CNVs in seemingly neutral regions can also affect gene expression and cause phenotypic variations [7]. Thousands of CNV-associated genes have been identified in our analysis, with some showing significant ethnic difference of CNV frequency. Our identification of 261 CNV genes with ethnic difference of CNV frequency between Caucasians and Asians provide us a better understanding that this structural chromosome variation may affect genes' expression in different ethnic populations and cause ethnic phenotypic diversity or ethnic specific diseases. Some identified CNVRs with frequencies greater than 1% were found to be overlapped with OMIM genes. Notably, our novel CNVR region_197, which has a frequency of 2% and includes copy number loss in Caucasian, overlaps with solute carrier family 25 gene (OMIM 212138) which is related to Carnitine-acylcarnitine translocase deficiency.

The Affymetrix GeneChip® Mapping 500K Array, which contains a series of high intensity probes, is an efficient tool for accurately determining micro-alterations in the human genome [4], [15], [17], [18]. Similarly, the Affymetrix GeneChip® Chromosome Copy Number Analysis Tool (CNAT) is software that has been widely used to detect sample level CNV calls and to estimate their boundaries based on CN states of SNP loci [19]–[21]. CNAT 4.0 contains CNAG algorithm same as the Copy Number Analyzer for GeneChip (CNAG) [22], which is another widely used CNV analysis software. CNV detection strategy in our analysis was referred from Zogopoulos et al [15], who used CNAG to detect CNVs in nearly 1,000 individuals of North American from Affymetrix GeneChip® Mapping 500K Array. They tested the sensitivity of CNAG algorithm on Affymetrix GeneChip® Mapping 500K Array by quantitative PCR. Together with previous reports [23], CNVs' frequencies in this study are accurate for those with occurrence lower than 5% and will underestimated about 1% for those with frequencies higher than 5%. The choice of reference sets can affect the sensitivity of CNV detection. 47±2 individuals from the same plate and the same ethnic group of our own data were used as reference sets. Utilizing sample data in the same plate as reference sets can avoid plate differences and yield better normalization.

We found that sample sizes can be an important factor in CNVR detection. Computer simulations were used to illustrate this issue. We randomly selected different numbers of individuals (50–900) from our CAU sample with 985 subjects and determined CNVR coverage rates across chromosomes. For each sample size, 100 resamplings were performed and average CNVR coverage rates were obtained and plotted by chromosome (**Figure S1**). The figure showed that increasing sample sizes yielded higher CNVR coverage rates. For example for 200 individuals, the coverage rates were 1.0–5.7% on autosomal chromosomes, and 10.4% on the X chromosome, compared to 4.1–10.7% on automosal chromosomes and 23.4% on X chromosome for 900 subjects. Our results on the X chromosome for 200 individuals is comparable, with an 11% coverage rate shown in a previous study using Hapmap subjects [4]. Utilizing the Affymetrix GeneChip Mapping 500K Array with samples of large size, provides opportunities for finding CNVs with higher resolution and increasing power. This should facilitate better descriptions of basic CNV characteristics, thereby enhancing our understanding of the role of CNV in the human genome and their differential role in different ethnic groups.

Our identification of nearly 3,000 CNVs and our characterization of CNV ethnic differentiation between Caucasians and Asians provide a better understanding of this structural chromosome variation. However, there are issues that remain to be addressed. For example, the accurate boundaries of CNVs have not been determined by current genotyping platforms; only two types of CNVs, “loss” and “gain”, are characterized and thus CNVs with different copy numbers may not be distinguished. Further, the relationship between CNVs and OMIM genes warrants further elucidation. Resolution of these issues, together with currently available knowledge on CNVs, will further elucidate the role of CNVs in several important biological phenomena, particularly, the contribution of genetic architecture to complex human diseases.

Genomic distribution and ethnic differentiation of CNVs have been characterized in our large random samples from Caucasian and Asian populations. Our findings of ethnic differentiation of CNVs, along with the newly created CNVR genomic map based on 1,677 Caucasian and Asian individuals, extend our understanding on the structural variation in the human genome and may furnish a basis for understanding the genomic differentiation of complex traits across ethnic groups.

## Methods

### Subjects

Subjects from two ethnic groups, US Caucasian (CAU) and Chinese Han (CHI), were included in this study. One thousand US Caucasian adult subjects, which consisted of 500 males and 500 females, were randomly selected from our established and expanding genetic repertoire currently containing more than 6,000 subjects. All Caucasian subjects lived in Omaha, Nebraska and its surrounding areas and were self-identified as being of northern European origin. They averaged 50.21±18.28 years in age, 1.71±0.10 m in height, and 80.13±17.80 kg in weight. Seven hundred Chinese Han adult subjects were recruited in Xi'an city and its vicinity in China, and consisted of 297 males and 403 females. They averaged 69.63±8.29 years in age, 1.59±0.08 m in height, and 58.00±9.88 kg in weight. All subjects were healthy and unrelated. The study was approved by the necessary Institutional Review Boards. Signed informed-consent documents were obtained from all participants before they entered the study.

### Microarray Hybridization and SNP Genotyping

Genomic DNA was extracted from whole blood either using a commercial isolation kit (Gentra systems, Minneapolis, MN, USA) according to the recommended protocol for CAU subjects, or using the phenol-chloroform extraction method for CHI subjects. DNA concentration was measured by spectrometry (DU530 UV/VIS spectrophotometer, Beckman Instruments, Fullerton, CA, USA). SNP genotyping with the Affymetrix Mapping 250K Nsp and 250K Sty arrays was performed at the Vanderbilt Microarray Shared Resource at Vanderbilt University Medical Center, Nashville, TN, using the standard protocol recommended by the manufacturer. Briefly, for each array, 250 ng genomic DNA was digested with either Nsp1 or Sty1, and ligated to adapters. A generic primer, recognizing the ligated adapter sequence, was used to amplify the ligation products in a PCR. The amplified DNA was assayed by agarose gel electrophoresis to verify an average size distribution of 250 bp to 1500 bp. The amplified DNA was purified per the manufacturer's protocol and was quantified by absorbance at 260 nm and 280 nm. 90 mg of purified DNA was cleaved using DNaseI and was visualized on a 4% agarose gel. Fragments less than 180 bp were hybridized to the appropriate array (Nsp or Sty). Arrays were stained with immunopure strepavidin (Pierce, Milwaukee, WI), washed by biotinylated antistreptavidin antibody (Vector Labs, Burlingame, CA), and scanned using R-phycoerythrin strepavidin (Invitrogen, Carlsbad, CA). Fluorescence intensities were quantified using an Affymetrix array scanner 3000-7G. Data management and analyses were performed using Affymetrix GeneChip® Operating System (GCOS). Genotyping calls, based on the fluorescence intensities, were determined using a dynamic model (DM) based algorithm and with a *p* value of 0.33 as well as the BRLMM algorithm [24], [25]. DM calls were used for quality control while BRLMM calls were used for subsequent CNV identification. Subjects with genome-wide SNP genotyping call rates less than 93% via the BRLMM algorithm, including 15 Caucasian subjects (6 males and 9 females) and 8 Chinese subjects (2 males and 6 females), were excluded from subsequent analyses.

### CNVs and CNVRs Determination

Affymetrix GeneChip® Chromosome Copy Number Analysis Tool (CNAT) Software V.4.0.1 was used to identify chromosomal copy number (CN) changes from fluorescence intensity data. CNAT includes a built-in probe-level quantile normalization of signal intensity, which is based on perfect match (PM) probes across the CEL files [26]. The genomic smoothing bandwidth was set as 100 kb. A Hidden Markov Model (HMM) based algorithm was used in CNAT for smoothing and segmenting CN data. There are five hidden states representing different biological phenomena: homozygous deletion (CN = 0), heterozygous deletion (CN = 1), normal diploid (CN = 2), single copy gain (CN = 3), and amplification (CN≥4). In our analyses, homozygous deletion and heterozygous deletion were defined as “loss”, whereas single copy gain and amplification were defined as “gain”. Default values in the CNAT program for the Affymetrix GeneChip® Mapping 500K Array were used for the HMM parameters (0.2 for the priors, 0.07 for standard deviation when CN equal to 2, 0.09 for other CN states, and 10 Mb for the transition decay value).

CNAT requires reference data sets to be compared with sample sets for estimating the CN state of each SNP locus. For CNV detection on autosomal chromosomes, samples on the same plate of 94 starting samples were assigned with equal probabilities into test and reference sets. This process yielded 47±2 subjects in a reference set in various sample plates and screened CN changes for all individuals. For CNV detection on the X chromosome, each subject was compared with a reference set comprising the remaining subjects of the same gender on the same plate. This resulted in 46±5 subjects for male or 47±4 subjects for female in a reference set in various sample plates.

The exact boundaries of CNVs cannot be obtained from data produced by the Affymetrix 500K SNP genotyping platform. However they can be approximated by physical positions (NCBI Build 35, May 2004) of the probe pairs having the maximal distance within a CNV, yielding conservatively shorter defined sizes of CNVs than the actual sizes. Individual CNVs were organized into CNV regions (CNVRs), which are stretches of genomic regions covering overlapping CNVs (**Figure S2**).

### Analysis of Ethnic Differentiation

Two important CNV characteristics, gains/losses and CN state frequency distribution, were used to study ethnic differentiation. For gain/loss differences, study subjects were categorized based on their ethnic groups (CAU versus CHI) and on the occurrence of CNV (gain, loss, no change). Numbers of study subjects in these categories were counted and were recorded by a 2×3 contingency table. Chi-square tests were then performed. These tests were carried out using chisq.test function in R version 2.6.0 (www.r-project.org).

### Population Substructure Detection

Detection of population substructure was carried out through the program STRUCTURE, which uses a Markov chain Monte Carlo (MCMC) algorithm to cluster individuals into different cryptic sub-populations [27]. For the program, non-admixture and correlated allele frequency models were selected, and the number of subpopulations was assigned as 2. 50,000 steps were run after a burn-in of 5,000. 2,000 unlinked SNPs were used.

## Supporting Information

Figure S1 (0.16 MB DOC)

Figure S2 (0.03 MB DOC)

Table S1 (0.48 MB XLS)

Table S2 (0.05 MB XLS)

Table S3 (0.20 MB XLS)

Table S4 (0.05 MB XLS)

Table S5 (0.31 MB XLS)

Table S6 (0.02 MB XLS)
