# Supplementary material for: Whole Genome Distribution and Ethnic Differentiation of Copy Number Variation in Caucasian and Asian Populations
Source: PLoS One. 2009 Nov 23;4(11):e7958. doi: 10.1371/journal.pone.0007958 (PMC2776354; doi:10.1371/journal.pone.0007958)
Supplement: Figure S1 — (0.16 MB DOC) [file pone.0007958.s001.doc]

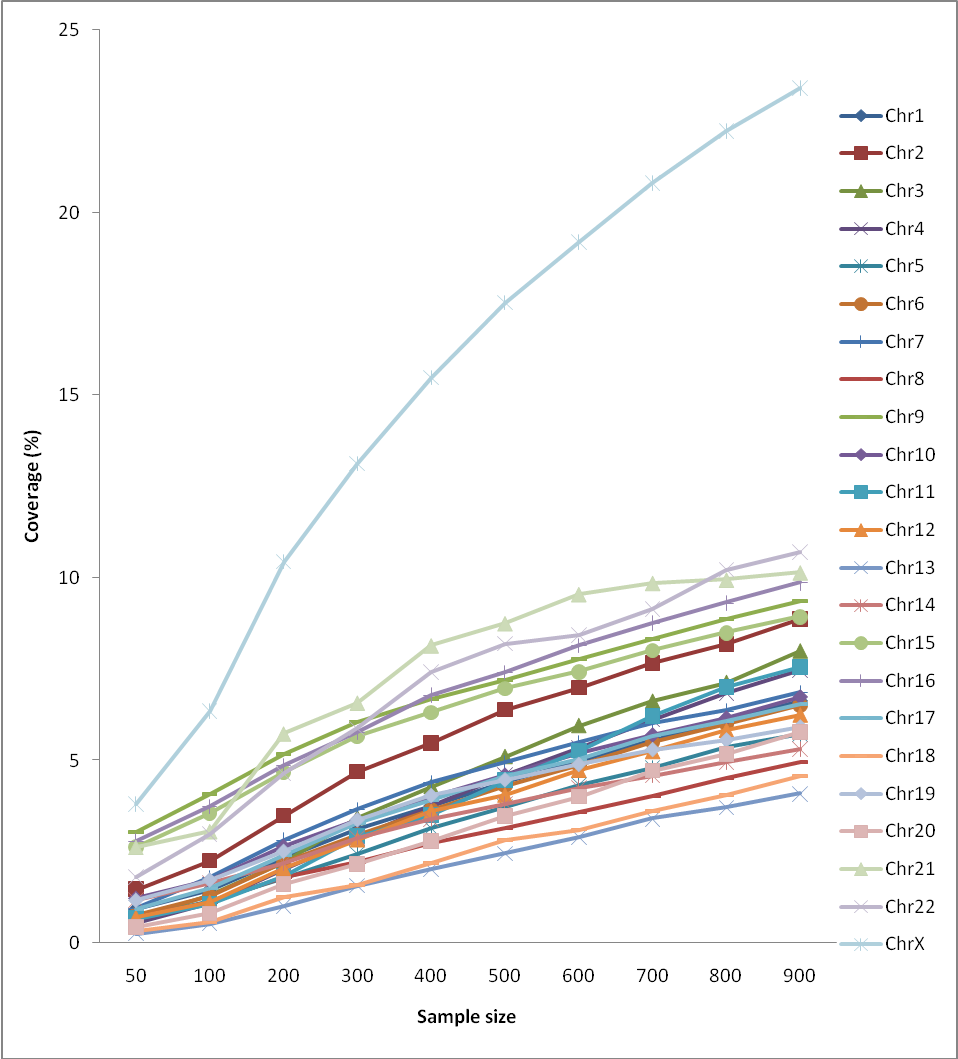


**Supplementary Figure 1.** Effects of sample sizes on CNVR identification in our Caucasian population with the Affymetrix Genechip® Mapping 500K Array. Sample size on x-axis indicates the number of individuals used in the analysis. Each data point is based on 100 random resamplings.
