# Supplementary material for: Whole Genome Distribution and Ethnic Differentiation of Copy Number Variation in Caucasian and Asian Populations
Source: PLoS One. 2009 Nov 23;4(11):e7958. doi: 10.1371/journal.pone.0007958 (PMC2776354; doi:10.1371/journal.pone.0007958)
Supplement: Figure S2 — (0.03 MB DOC) [file pone.0007958.s002.doc]

Sample 1

Sample 2

Sample 3

Sample 4

500K SNPs

Chromosome

CNVs

CNVRs

**Supplementary Figure 2.** Examples of copy number identification by CNAT using Affymetrix 500K SNP Mapping Array data and the relationship among SNPs, CNVs, and CNVRs. An illustration of the relationship among SNPs, CNVs, and CNVRs. Briefly, CNVs are bounded by Affymetrix 500K SNPs, and CNVRs are genomic regions covered by overlapping CNVs.
